# High frequency synchrony in the cerebellar cortex during goal directed movements

**DOI:** 10.3389/fnsys.2015.00098

**Published:** 2015-07-21

**Authors:** Jonathan D. Groth, Mesut Sahin

**Affiliations:** Department of Biomedical Engineering, New Jersey Institute of TechnologyNewark, NJ, USA

**Keywords:** cerebellar oscillations, cerebellum, paramedian lobule, multi-electrode arrays, phase synchrony

## Abstract

The cerebellum is involved in sensory-motor integration and cognitive functions. The origin and function of the field potential oscillations in the cerebellum, especially in the high frequencies, have not been explored sufficiently. The primary objective of this study was to investigate the spatio-temporal characteristics of high frequency field potentials (150–350 Hz) in the cerebellar cortex in a behavioral context. To this end, we recorded from the paramedian lobule in rats using micro electro-corticogram (μ-ECoG) electrode arrays while the animal performed a lever press task using the forelimb. The phase synchrony analysis shows that the high frequency oscillations recorded at multiple points across the paramedian cortex episodically synchronize immediately before and desynchronize during the lever press. The electrode contacts were grouped according to their temporal course of phase synchrony around the time of lever press. Contact groups presented patches with slightly stronger synchrony values in the medio-lateral direction, and did not appear to form parasagittal zones. The size and location of these patches on the cortical surface are in agreement with the sensory evoked granular layer patches originally reported by Welker's lab (Shambes et al., 1978). Spatiotemporal synchrony of high frequency field potentials has not been reported at such large-scales previously in the cerebellar cortex.

## Introduction

The cerebellum is essential to sensory-motor integration for fine control of motor performance. It has also been shown to be important in many cognitive tasks of the brain (Schmahmann, 2010). There are two main afferent pathways to the cerebellum. The first is the mossy fiber pathway which originates from a number of cerebral areas and the spinal cord and terminates in the granular layer of the cerebellar cortex where they synapse onto the granule cells, which then synapse onto the Purkinje cells (Eccles et al., 1967; Ito, 2010). The second is the climbing fiber pathway that originates in the inferior olive and projects directly onto the Purkinje cells (Voogd et al., 1981; Buisseret-Delmas and Angaut, 1993; Voogd and Glickstein, 1998; Sugihara et al., 1999; Apps and Garwicz, 2005). The different input pathways and their termination on the Purkinje cells form the basic processing unit in the neural circuitry of the cerebellar cortex. The sensory mossy fiber inputs to the granular cells form a patchy architecture (Shambes et al., 1978) while the climbing fiber inputs form microzones oriented in the parasagittal direction (Garwicz et al., 1998a,b). These two architectures are believed to be functionally overlapping in what is called the one map theory (Apps and Hawkes, 2009; Cerminara et al., 2013). Current understanding of the cerebellar function is mostly built upon the cerebellar anatomy and single cell recording data collected using indwelling electrodes. Cellular level neural signals are important to understand how the cerebellar circuitry processes information. On the other hand, multi-channel field potential recordings in behaving animals can provide direct evidence on the cerebellar function at a larger scale.

The cerebellum is known to express several types of oscillations arising from different cortical layers. Oscillatory activity is an important part of neural function and is present throughout the brain. Oscillations have been reported to help shape the timing and binding of the actions of neurons into a cohesive representation in both local circuits and across regions in the brain (Buzsáki and Draguhn, 2004; Buzsaki, 2006). These rhythms are necessary to create a unified picture of the world and motor responses (Buzsaki, 2006).

Two oscillations that have recently garnered interest in the cerebellum are the very slow (~1 Hz, see Ros et al., 2009) and the 4–25 Hz oscillations that originate in the granule cell layer (Pellerin and Lamarre, 1997; Hartmann and Bower, 1998; D'Angelo et al., 2001; Courtemanche et al., 2002). The granule cells also show a resonance in the 4–10 Hz range (D'Angelo et al., 2001). The Golgi cells of the molecular layer show a resonance and pace making in the same range, however, it has been argued that the granule cell resonance may be independent of the Golgi cell pacemaking (Solinas et al., 2007a,b; D'Angelo et al., 2013). The 4–25 Hz oscillations show a strong similarity to the granular layer multi-unit activity, a weaker one to the Purkinje cell activity (Courtemanche et al., 2002; Lu et al., 2005), and no correlation to the climbing fiber activity (Courtemanche et al., 2002).

Another major oscillation type found in the cerebellum is the fast oscillations. The high frequency cerebellar oscillations in the 150–250 Hz range were first discovered by Adrian in 1934 (Adrian, 1934). It was later shown that the fast frequency components are related to the state of the animal (Oehler et al., 1969). They appeared during preparation for goal directed movements but not during non-goal directed repetitive movements. These high frequency field potentials have been shown to originate from the Purkinje cell layer and are believed to be the result of Purkinje cell simple spike synchrony (Cheron et al., 2004, 2008; de Solages et al., 2008; Middleton et al., 2008). There are several theories as to how this spike synchrony arises. One theory is that the synchrony is caused by common inputs such as the mossy fibers (Heck et al., 2007). Another theory is that the synchrony is caused by the inhibitory Purkinje axon collaterals (de Solages et al., 2008). The most probable case is that it is a combination of multiple influences.

Purkinje cell synchrony may play an important role in cerebellar information processing. There is evidence that the cerebellum may encode information in the spatiotemporal domain (Isope et al., 2002; De Zeeuw et al., 2011). It has recently been shown that synchronous inhibition of the deep cerebellar nuclei (DCN) by the Purkinje cells can lead to rebound firing of the DCN cells, which can modulate the motor output (Witter et al., 2013). Therefore, synchronous spiking of Purkinje cells has been proposed to play an important role in cerebellar signal processing and thus the cerebellar function.

For these reasons an investigation of high frequency field potentials in the cerebellar cortex could provide important clues to the function of and information processing in the cerebellum. However, these cerebellar oscillations have not been as extensively studied as in other areas of the brain such as the hippocampus. In this study we investigated the spatio-temporal patterns of high frequency synchrony in the cerebellar cortex during goal directed movements in order to better understand their functional role. We employed chronically implanted μ-ECoG electrode arrays for the first time to capture the field potentials from the cerebellar surface without penetrating the cortex. The use of a μ-ECoG array allowed us to study synchrony between 31 different sites covering relatively a large area of the PML cortex in the same preparation. Chronic implantation of the array also made it possible to record these signals in behaving animals in multi-day trials for a few weeks.

## Methods

### Animal training

The animals were trained for the lever pressing task prior to implant surgery. The lever was attached to a robot with haptic feedback (Falcon, Novint Inc., Albuquerque, NM) that simulated a viscous environment with programmable parameters via the computer. A food reward (sugar pellet) was delivered through a tube located a few centimeters to the left of the lever once the lever was pressed. The animals were placed on food restriction a few days prior to training. During training animals were allowed to freely roam the cage. The trial was initiated by a computer-generated beep sound. After the beep the animals could press the lever any time within the following 5 s window. The 5 s data segment containing the lever press in the middle of the episode was saved by the computer for later analysis. The rats learned this behavior usually within a week by training about 30 min each day. The proximity of the lever to where the sugar pellet drops sometime allowed the rats to rest the arm on the lever while picking the sugar pellet with their mouth. The trials that did not start with the hands on the floor were excluded from the analysis.

### Implant surgery

**Figure 1:**
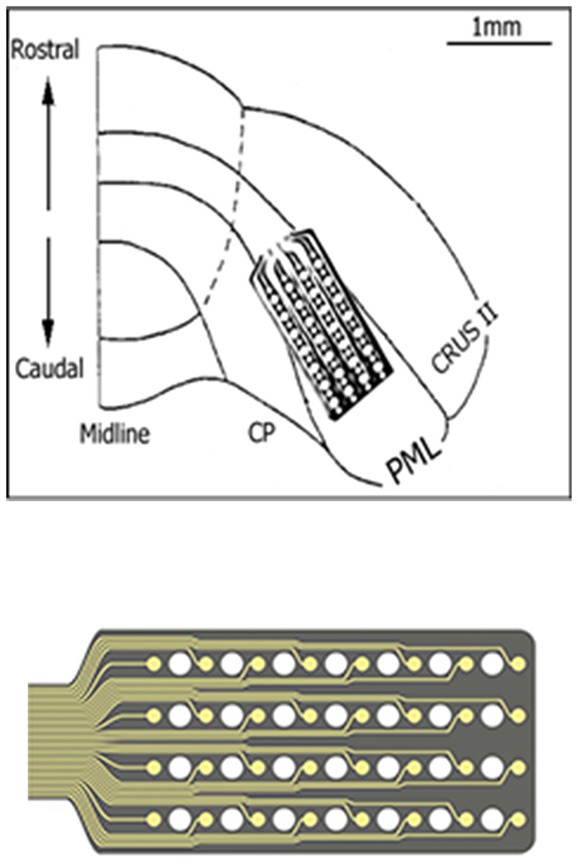
**The implant location of the recording array is depicted on the right paramedian lobule (PML) of the posterior cerebellum**. The 4 × 8 arrangement of the contacts on the micro-electrode array is also shown at a large scale on the bottom. A few contacts fell outside the paramedian lobule because of medially narrowing shape of the PML.

Micro-ECoG arrays were chronically implanted in three Long Evans rats (350–450 g) using sterile surgical techniques. All procedures were approved and performed in accordance to the guidelines of the Institutional Animal Care and Use Committee, Rutgers University, Newark, NJ. The rats were anesthetized with ketamine and xylazine (100 and 10 mg/kg, respectively, IP) and additional doses were administered as needed during the surgical procedure. The skull over the right paramedian lobule (PML) of the cerebellum was removed and a “T” shape cut was made into the dura using fine scissors. A custom-design, flexible substrate (12 μm polyimide), 32-contact electrode array (NeuroNexus, MI) was placed subdurally by sliding it through the dural cut on the right paramedian cortex with the medial edge of the electrode positioned about 1 mm away from the paravermal vein and fixed in place with very small amounts of cyanoacrylate (gluture, WPI Inc.) at four corners to the cortical surface (Figure 1). Electrode contacts were 50 μm in diameter and located 300 μm apart from each other in a 4 × 8 configuration, hence covered approximately 900 × 2100 μm of the PML in the medio-lateral orientation as shown in Figure 1. The dura was sealed with a fast curing silicone elastomer (Kwik-Cast, WPI Inc.) over the array. A stainless steel wire that served as a reference electrode was laid down over the area on the backside of the array and secured in place with cyanoacrylate. The Omnetics micro connector at the end of the ribbon cable was fixed on the skull using dental acrylic and five stainless steel screws. The recordings started about a week after surgery with a 31-channel head-stage amplifier (Gain = 100, TBSI, NC) inserted into the micro connector on the head while animals were placed in a large Faraday cage. The signals were sampled at 20 kHz in 5 s episodes containing the behavior.

### Data analysis

All data analysis was performed using Matlab (Mathworks, Natick, MA). The power spectrum was calculated using the multitaper method for each trial and channel using the Chronux toolbox (Bokil et al., 2010). Power spectra were then averaged across all trials and channels. The spectrograms were also calculated using multitaper method with a window length of 200 ms and a step size of 100 ms. The inter-contact coherence spectra were computed and averaged between all channel combinations in each trial using Matlab's *mscohere* function (Hamming window, 50% overlap), which is a magnitude squared coherence estimate with values between 0 and 1 that indicate how well one signal corresponds to another at each frequency. The coherence is a function of the power spectral densities, *P*_*xx*_(*f*) and *P*_*yy*_(*f*), of *x* and *y*, and the cross power spectral density, *P*_*xy*_(*f*), as given below:

$$\begin{array}{rc} C_{xy}\left(f\right)=\frac{{\left|P_{xy}\left(f\right)\right|}^{2}}{P_{xx}\left(f\right)*P_{yy}\left(f\right)} & \end{array}$$

In order to produce the coherogram plots, i.e., coherence as a function of time and frequency, the inter-contact coherence spectra were computed within a 200 ms window (also Hamming windowed with 50% overlap) that was shifted in 100 ms steps for the entire duration of the trial.

For phase synchrony we developed our own Matlab code based on the analysis method described in Tass et al. (1998). In brief, the phase information in each neural channel was found from the analytic signal of the Hilbert transformation as a function of time in a running window. The Hilbert transform and the phase signal are computed by the following equations respectively.

$$\begin{array}{l} HT\left(x\right)\left(t\right)=\frac{1}{\pi }*CPV\int_{-\infty }^{\infty }\frac{x\left(\tau \right)}{t-\tau }d\tau \\ \;Ax\left(t\right)=x\left(t\right)+jHT\left(x\right)\left(t\right) \end{array}$$

Where *x*(*t*) is the signal to be transformed and *HT*(*x*)(*t*) is the Hilbert transform of *x*(*t*). CPV represents the Cauchy principle value method of integration. The phase signal is represented by *Ax*(*t*). The differential phase between channels was computed for each channel pair in a running window over time. Shannon entropy was computed and used as a synchrony measure from the statistics of inter-channel synchrony given the assumption that two signals are synchronous for a given time window when their differential phase is approximately constant. The Shannon entropy index was given by:

$$\begin{array}{r} \gamma =\left(Hmax-H\right)∕Hmax \end{array}$$

with the entropy (*H*) given by:

$$\begin{array}{r} H=\overset{N}{\underset{k=1}{\sum }}pk*ln\left(pk\right) \end{array}$$

The value of *N* is the number of phase bins defined by *N* = exp[0.626+0.4^*^ ln(M-1)] where M is the number of time samples within the running window (200 ms). The value *p*_*k*_ is the relative frequency of differential phases within the *k*^*th*^ bin. *H*_*max*_ is defined as *H*_*max*_ = *ln*(*N*). Synchrony values (γ) of 0 and 1 meant no synchrony and perfect synchrony, respectively.

In addition, clustering analysis was performed on the inter-contact synchrony values to determine which channels followed a similar trend as a group around the time of lever press and if there was a spatial pattern to the synchrony. The synchrony values were averaged across the frequency band of interest (150–350 Hz) between all contact pairs during lever press in multiple trials. A long vector was formed for each contact that consisted of its synchrony values with all the other contacts as a function of time (11 time points at 50 ms intervals) and in multiple trials. In other words, each vector contained many small vectors of 11 synchrony values, concatenated with other small vectors resulting from multiple trials and with multiple contacts. This way all the variation across the trials was contained in one large vector by combining the inter-contact synchrony data from all trials. The synchrony vectors from all 31 contacts were then clustered using the k-means method in Matlab. Since the clusters were observed to change between clustering attempts, one thousand clustering iterations were implemented with randomized starting points. This accounted for the variations in clustering due to the dependency of the algorithm on the starting points. The success rates of clustering together were calculated between each contact pair. Contact pairs that ended up in the same cluster in greater than or equal to 95% of the attempt were considered to be in the same group. After the final groups were derived, the centroids of the contact groups (i.e., their vectors) and Euclidian distances from the centroid to each vector were calculated. The strength of membership for each contact to a group was calculated using the following equation.

$$\begin{array}{r} SM=1-\frac{D1}{\frac{\sum Dn}{N}} \end{array}$$

Where SM is the strength of membership for which 1.0 is the highest value and lower values represent weaker membership to a group. D1 is the Euclidian distance of a contact to the centroid of its own group. The numerator represents the average distance of a contact to the centroids of all the groups.

ANOVA analysis was performed on the average synchrony values for each time point. The synchrony values for all contacts were averaged for each trial. The values for each of the 11 time points were compared using ANOVA analysis for each animal. If the ANOVA analysis showed that there was a statistical significance between the time points a Turkey–Kramer test was performed on the data to determine the significance between the individual points. The time point pairs, (−0.25, −0.1 s), (−0.1, 0 s), and (0, 0.1 s), were compared for significant difference for each animal.

## Results

**Figure 2:**
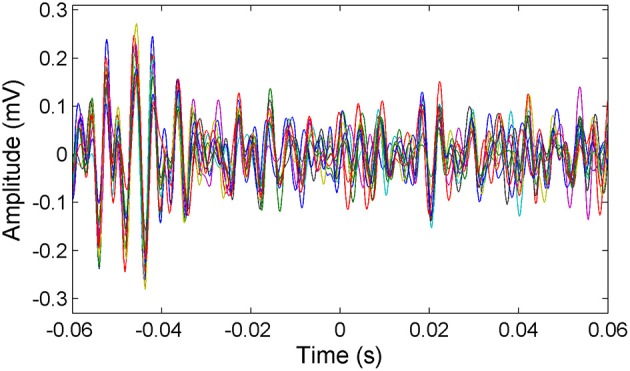
**Representative multi-channel cerebellar signals plotted (only 16 channels for clarity) around the time of lever press (*t* = 0)**. Signals are filtered between 150 and 350 Hz to show the frequency band of interest. Multi-channel signals go in and out of synchrony episodically.

**Figure 3:**
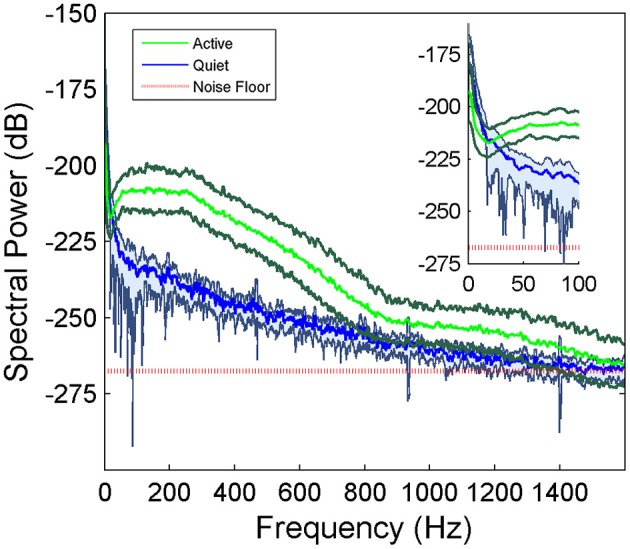
**Average power spectra of the cerebellar signals collected from a rat during active behavior involving the forelimbs (*N* = 42 trials of 5 s recordings) and quietly resting (*N* = 5 trials) episodes in the same recording sessions**. Shaded areas indicate standard deviations. There is a large difference between the active and quietly resting states especially below 900 Hz. The red line indicates the estimated noise floor above which the signal power is considered to be from neural origin.

μ-ECoG signals of the PML were analyzed during a lever pressing task. The high frequency components (150–350 Hz) were present in all recordings and showed oscillatory patterns with rapidly changing amplitudes during the movements of the animal (Figure 2). However, a direct correspondence between the instantaneous signal amplitudes and the motor behavior was not observed. The multi-channel signals were seen to move in and out of synchrony in short episodes. Those episodes with high inter-contact synchrony tended to have larger amplitudes since synchronous field potentials add constructively at the recording sites in a volume conductor. The neural signal power spectrum typically extended up to 900 Hz (active plot in Figure 3) beginning to drop only after 300 Hz, and a dip was observed with its center in the beta frequency range (inset). The power spectra of these 5 s long recording epochs did not have a sharp peak, which would indicate oscillations at a single or a narrow band of frequencies. There was a clear difference in signal power between the active and quiet states of the animals, defined as quietly sitting in the cage in awake state with no visible movements.

**Figure 4:**
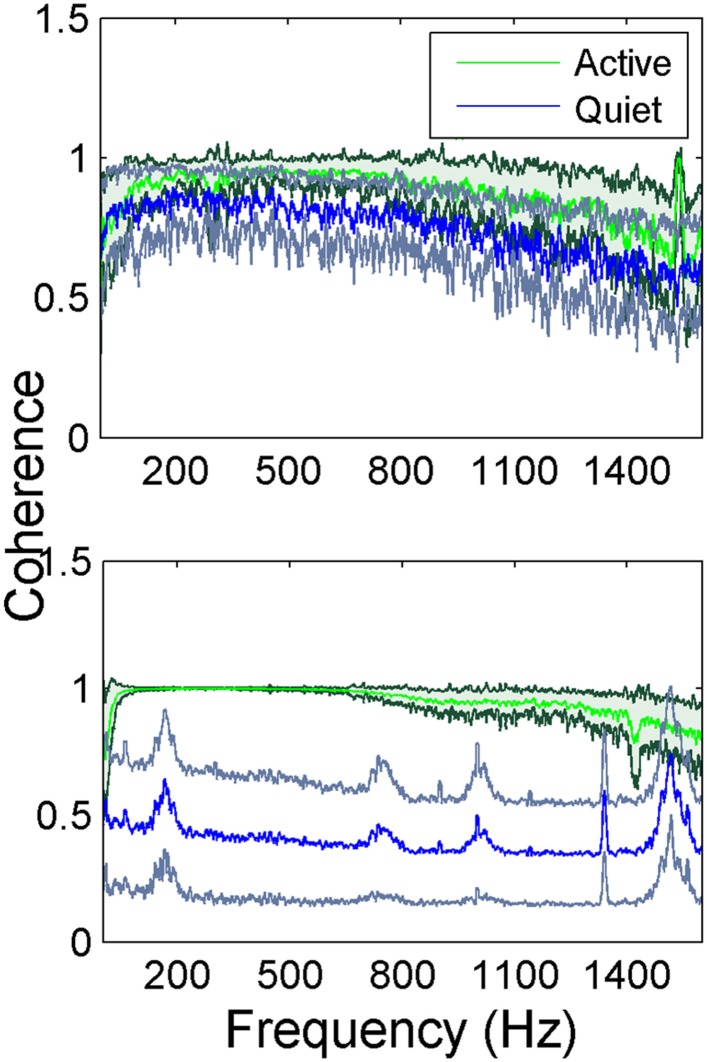
**Average coherence between all channel pairs in the electrode array in two rats while the animals are performing lever press**. In animal 1 (**top figure**) the green trace (dark green standard deviation) represents the coherence during the lever press task and the blue trace (gray standard deviation) is the quiet state (a total of *N* = 42 trials for lever press and *N* = 5 trials for the quiet state). Animal 2 (**bottom figure**) the green trace (dark green standard deviation) represents the coherence during the lever press task and the blue trace (gray standard deviation) is the quiet state (a total of *N* = 23 trials for lever press and *N* = 5 trials for the quiet state). The small peaks at higher frequencies are most likely artifacts produced by the coherence function between very small amplitude noisy signals.

The inter-contact coherence during lever press (active in Figure 4) had near maximum values for the entire range of frequencies where there was neural signal power. The fact that coherence was high even at high frequencies where the signal power was very small (>900 Hz) suggests similar temporal signal patterns between recording channels at millisecond resolution. The coherence plots of the so called quiet episodes had varying amplitudes depending on the state of the animal that we were not able to judge visually, but in general they were much smaller compared to the active case at all frequencies. The quiet spectra had distinct peaks when the coherence values became lower as observed in animal two (bottom plot in Figure 4). Those peaks above 900 Hz must be artifacts produced by the coherence function between very small amplitude noisy signals. The peak between 100 and 200 Hz, however, represents converging of neural signals into a narrower band during the resting state of the animal.

**Figure 5:**
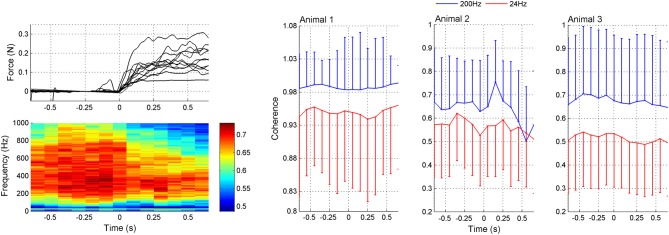
**Coherogram data during lever press**. Upper left plot: Representative force traces applied to the lever by the rat's forearm. Only a subset of the trials are plotted for clarity. The lever press takes place while the force is increasing and then the forearm rests on the lever while it is arrested at a fixed position by the computer. Lower left plot: Average coherence between all channel pairs in multiple trials (*N* = 42 trials from three rats) as a function of time in a 200 ms window leaping in 100 ms steps. There is a peak in the lower band around 24 Hz. The high band extends from about 100–800 Hz, though the maximum power levels during the movement, initiated at 0 s, is found between 200 and 350 Hz. Right plots (Animals 1–3): Coherence values specifically at 24 and 200 Hz as a function of time. The mean ± std are shown at both frequencies from the same trials (*N* = 42, 23, and 18 trials in animals 1–3, respectively). Either positive or negative std is not shown in each plot for clarity.

The coherogram method was employed to determine how the inter-channel coherences varied as a function of time. The coherogram plot (Figure 5) demonstrated broadening of the frequency band and increasing in strength just before the lever press (around *t* = −0.2 s). After the initiation of the lever press (*t* = 0), there was a drop in coherence followed by a slight increase again. There is also a peak of coherence in the beta band (~24 Hz, green stripe) that follows a similar temporal trend with the high frequency band coherence (plots to the right in Figure 5). The correlation between the mean values of the 24 and 200 Hz coherence plots as a function of time within *t* = ±0.6 s suggests a high degree of similarity (*R* = 0.70, 0.55, and 0.72 for animals 1–3, respectively). Based on the coherograms in Figure 5, we focused the synchrony analysis into the 150–350 Hz frequency band where the highest coherence values were detected before and after the behavior.

**Figure 6:**
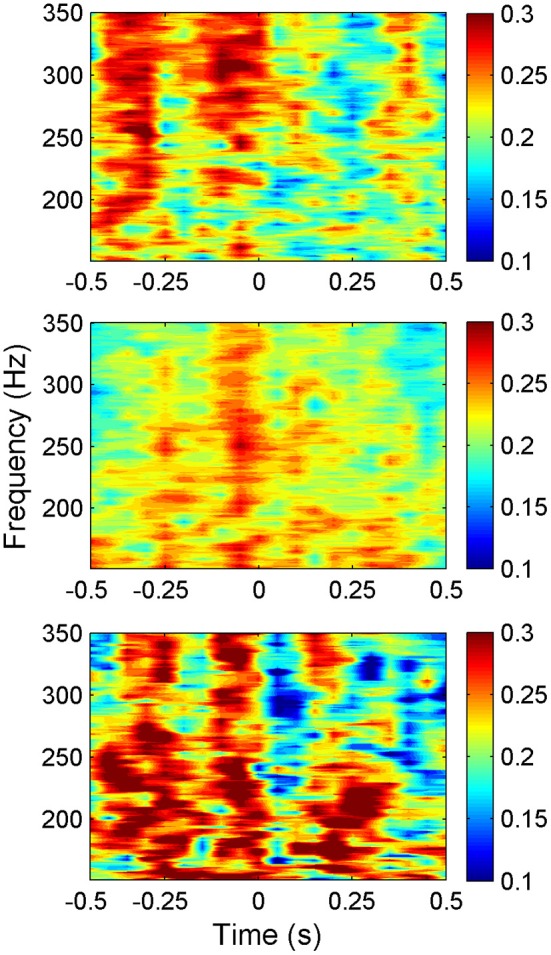
**Synchrony values plotted as a heat plot within the frequency band of interest (150–350 Hz) as a function of time for all three animals (*N* = 42, 23, and 18 trials top to bottom respectively) showing a consistent pattern of synchronization between animals around the time of lever press**. The pattern shows an increase in synchronization just before the movement, then a sharp decrease at the initiation of the lever press (*t* = 0). Note that the alternating bands of high and low synchrony exist with or without behavior outside the time window shown.

The average synchrony plot from multiple trials in each animal (Figure 6) showed that there were alternating bands of high and low synchrony in time. There was a great deal of similarity between all three animals in temporal variation of synchrony values around the time of lever press. In all animals, a period of high synchrony occurred just before the initiation of the lever press. At the time of lever press initiation (*t* = 0), there was a drop in synchrony followed by an increase again, similar to the trend in signal power. The times of second peaks in synchrony after lever press initiation closely correlated with transition times in the lever force plot from rising to plateau.

**Figure 7:**
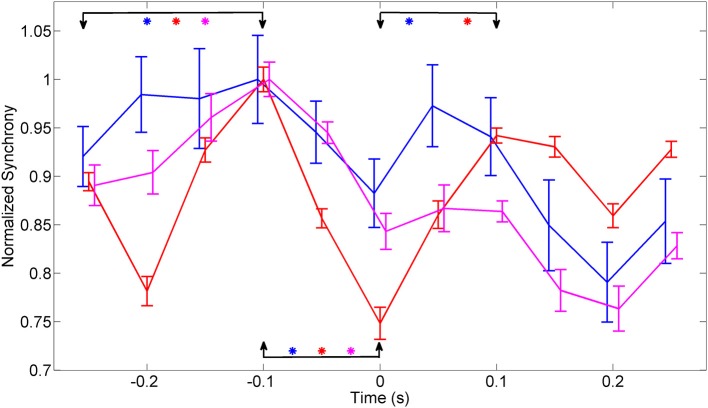
**Synchrony values were averaged across 150–350 Hz band, normalized to eliminate inter-contact variations, and averaged across contacts and trials in each animal (*N* = 42, 23, and 18 trials in animals 1–3, respectively)**. The synchronization pattern is similar in all three animals around the time of movement initiation. Bars indicate overall standard deviation from each animals. The arrows mark the channels that were compared, using ANOVA analysis, for statistical significance and the stars represent significant difference for each animal represented by the line color with animal 1 (Blue), animal 2 (red), and animal 3 (magenta). There is a significant difference in all three animals between time points (−0.25, −0.1 s) and (−0.1, 0 s). The time points (0, 0.1 s) showed a significant difference in only animal 1 and animal 2.

The plots in Figure 7 were obtained by merging the amplitudes of synchrony values across all the frequencies within the entire band of 150–350 Hz from multiple trials in each animal. ANOVA analysis showed a significant difference between the synchrony values in all three animals between time points (−0.25, −0.1 s) and (−0.1, 0 s) relative to the initiation of the lever press (α = 0.01). There was significant difference between the time points (0, 0.1 s) in animal 1 and animal 2.

**Figure 8:**
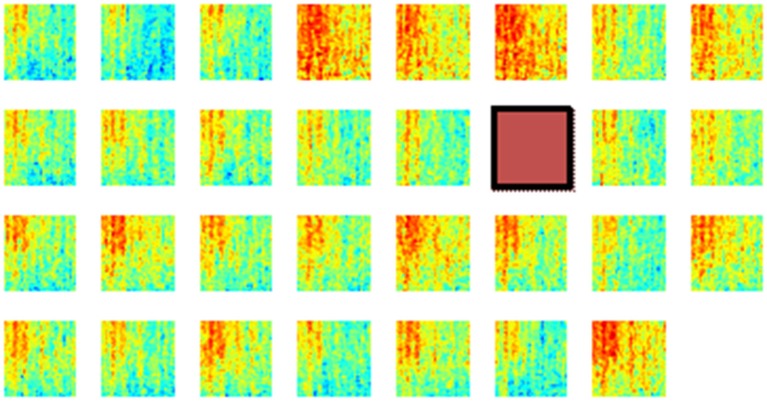
**Phase synchronies between a reference contact (brown square) and all the others in one rat during multiple lever pressing trials (*N* = 40)**. These plots show that the synchronization spread over the entire area covered by the array, although the highest synchrony values are to be found near the reference contact. Synchrony values are not monotonously decreasing with distance from the reference electrode. The X-Y axes for each mini plot are same as in Figure 6. A channel is missing at the corner because of one less count on the number of amplifier channels.

Next, the spatial distribution of phase synchrony over the recording area was analyzed. An example of the synchrony for each contact in the array with respect to a reference contact (red square) is shown in Figure 8. In all animals, synchrony could be seen spread over the entire cortical area covered by the electrode array. The highest synchrony was observed generally with contacts near the reference contact against which all the synchrony values were computed. However, not all the contacts next to the reference showed high synchrony. The synchrony values did not vary monotonously by distance as it would be expected if the synchrony was due to a distant common source, e.g., cortical cells in deep sulci or deep cerebellar nuclei. Therefore it was inferred that the synchrony between the recording channels emerged from the cortical neurons beneath the electrode and not from a distant, common mode signal.

**Figure 9:**
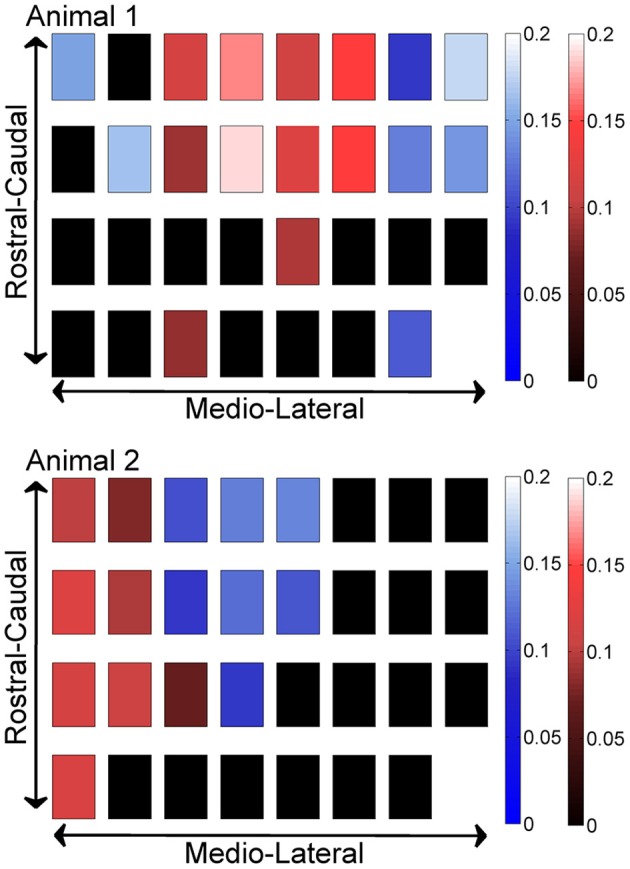
**Clustering of contacts by synchrony in two different animals (*N* = 42 and 23 trials), with each square representing an electrode contact**. The two largest clusters are marked by two different contact colors (red and blue) in each animal. The color gradients represent the strength of membership (SM) for each contact to its own group. The smaller the value, the weaker the membership. The groups present a patchy spatial organization across the PML cortex, rather than parasagittal zones.

**Figure 10:**
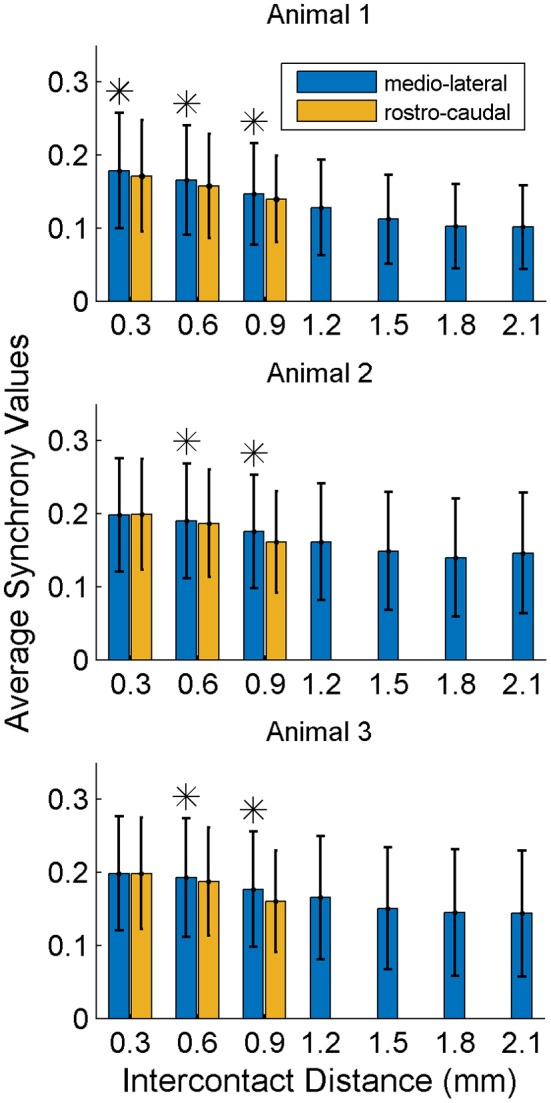
**Synchrony values as a function of inter-contact distance and orientation**. The synchrony on average decreases slightly by distance in both directions. Medio-laterally oriented contacts (in blue) have a slightly higher (^*^significance, *p* < 0.01) synchrony than rostra-caudal ones (yellow) for inter-contact distances of 0.6 and 0.9 mm in all three animals, and also for 0.3 mm in the first rat. Other inter-contact separations in the rostra-caudal direction are not available for comparison. The number of trials in each animal are the same as in Figure 8.

In order to investigate if some contacts synchronized and followed a similar synchrony trend in time as a group, we applied the clustering analysis to the synchrony values from each contact (or recording channel) as a function of time (see Methods). The clustering of contacts from two different animals is presented in Figure 9. The plots represent the two largest clusters for each animal where the membership strengths (SM) are color coded. The contacts within the same group are contiguous in the bottom plot but not in the top (blue group is divided). There does not appear to be any directional orientation in synchrony in these maps. A statistical analysis, however, suggests slightly higher synchrony values in the medio-lateral direction (Figure 10). This analysis also clearly demonstrates that the synchrony values decrease very slowly as a function of inter-contact distance.

## Discussion

The lever pressing task was chosen because it provided a non-cyclic, stereotypical behavior that presumably involved the cerebellum and the PML in particular. Difficulty arises in interpretation of neural signals collected during cyclic motor tasks in terms of their temporal relation to the behavior, such as face cleaning. Lever pressing task has been shown to give rise to high frequency oscillatory activity in the cerebellum (Oehler et al., 1969).

We can only speculate about the source of the neural signals recorded from the cerebellar surface. The single spike activity of the Purkinje cells is estimated to have the largest share in the signals due to their proximity to the surface, although granular cells and their ascending axons may be contributing as well. Our results on coherence and synchrony are in agreement with those reports on characteristics of Purkinje simple spike activity recorded with indwelling electrodes, as discussed below. Parallel fiber action potentials cannot be considered as a significant source for surface field potentials because of large variability in their times of arrival under each electrode contact and thereby making their constructive superposition highly unlikely. The post synaptic potentials of the Purkinje dendrites and mossy fiber terminals can also be ruled out as a major source due to the low frequency content of their activity compared to the somatic spikes.

We first investigated the amplitude modulation of the high frequency oscillations during the lever press task. The analysis showed that the high frequency oscillations (150–350 Hz) increased in power before the lever press, decreased during the lever press, and increased again after the lever press was completed. This temporal trend of neural activity in relation to a motor function is in agreement with reports on Purkinje cell single spike activity. Abrupt changes in simple spike frequency at the time of rat's hand touching the food pellet were reported from the on-beam and off-beam Purkinje cells of the PML (Heck et al., 2007). Purkinje cell spike frequencies also increased during forelimb swing compared to the stance phases of walking in cats (Armstrong and Edgley, 1984). Similarly, in rhesus monkeys, the Purkinje cells increased their firing rates during brief, cue-initiated movements (Thach, 1970a,b).

Interestingly our coherence analysis also indicated that there were similar trends in the high frequency coherence and the beta band coherence amplitudes in relation to the lever press. This trend of high frequency band coherence time-locked to the behavior was similar to that of 4–25 Hz band signals of the cerebellar cortex, which have been shown to emanate from the granule layer (Pellerin and Lamarre, 1997; Courtemanche et al., 2002). This suggests that the granule layer oscillations and the high frequency oscillations observed from the pial surface in this study may be related. Beta band oscillations of the cerebellum and that of the motor cortex were coherent during sustained movements in monkeys (Soteropoulos and Baker, 2006).

Temporal analysis showed alternating bands of high and low inter-contact phase synchrony throughout the recording episodes. There was a consistent pattern in synchrony that was time-locked to the moment of lever press across all trials. The inter-contact synchrony values peaked just before the movement, took a sharp decline at the time of lever press, and presented a smaller peak after the lever press was completed. This pattern was reproducible in all three animals. These results strongly argue that the spatiotemporal synchrony in the high frequencies (150–350 Hz) of the PML field potentials are related to the forelimb behavior. The heightened simple spike synchrony for a few hundred milliseconds before the hand touching the food pellet was interpreted as the involvement of the PML in reaching behavior (Heck et al., 2007). This type of zero-lag simple-spike synchrony was significantly higher in the medio-lateral direction along the parallel fibers (on-beam) compared to the rostra-caudal (off-beam) orientation of the recording electrodes at 305 and 610 μm contact separations. The spatial extent of the field potential synchrony reported here is much larger than what was seen in the synchronization of Purkinje cell single spike activity and only slightly dependent on direction. In many studies under several different anesthetic regimens Purkinje cell single spike synchrony (~1 ms) was found between cells separated by less than 100 μm (Bell and Grimm, 1969; MacKay and Murphy, 1976; Ebner and Bloedel, 1981; De Zeeuw et al., 1997; Shin and De Schutter, 2006). Others have reported synchronizations up to a few hundred micrometers and quickly decreasing by distance (Heck et al., 2007; de Solages et al., 2008). The field potential phase synchrony measures reported here spread across the entire electrode array (0.9 × 2.1 mm) and did not show a strong dependency on distance. Such a large scale synchrony, clearly detectable as a field potential in the PML must be representing a more global and perhaps centrally driven state, such as an elevated level of attentiveness, rather than encoding a specific behavior. Our results do not argue against the factors that may be responsible for simple spike synchrony. Instead, there may be two different mechanisms playing a role at two different scales; axon collaterals augmenting the micro scale synchrony at distances of a few hundred micrometers (Heck et al., 2007; de Solages et al., 2008) and the mossy fiber inputs modulating this synchrony at larger scales as seen in this study.

The clustering analysis showed only a slight directional preference in spatial distribution of synchrony. The size and location of the synchronized areas are consistent with the patchy organization of the granule layer to tactile stimulation, as originally reported by Welker's lab (Shambes et al., 1978), rather than the parasagittal zones formed by the climbing fiber system. The size of the cortical area investigated was still small to observe multiple patches within the same group of synchronized contacts. It was originally hypothesized by Llinás that ascending segments of granular cell axons preferentially terminate on the superjacent Purkinje cell dendrites (Llinás, 1982). Ekerot and Jorntell found that the receptive fields of Purkinje cells corresponded to that of the mossy fiber terminal aggregates rather in neighboring microzones (Ekerot and Jorntell, 2003). The microzonal relation was preserved more strictly between the receptive fields of the mossy fiber terminal aggregates and the molecular layer interneurons. Thus, considering these reports collectively there may be a modulation effect of the Purkinje simple spike activity within a cortical area by the granular cells subjacent roughly to the same area, even though this spatial correspondence may not be at the resolution of individual microzones.

Finally, we speculate that the sharp drop in synchrony at the time the rat's hand touches the lever must be due to the stimulation of the cutaneous mechanoreceptors. It has been argued that the peripheral inputs entrain and synchronize the Purkinje simple spikes and thereby modulate the output of the deep cerebellar nuclei (see Person and Raman, 2012 for review). That is, higher synchrony is associated with increased cerebellar output. Again these seemingly controversial ideas can be reconciled by making a distinction between simple spike synchrony of local Purkinje cells and the large scale synchrony of the field potentials recorded in this study. The cutaneous inputs must be entraining various subpopulations of Purkinje cells at different frequencies and therefore desynchronizing these groups of cells from each other whereas they are all driven at the same frequency before the stimulus by a common input through the mossy fiber system. This is supported by the fact that the coherence spectra had a distinct peak at ~100–200 Hz in a quietly resting animal which was replaced by a wider spread high coherence values during activity. A slight broadening of the field potential spectral peak was observed in the awake rat cerebellum as compared to anesthetized preparations (de Solages et al., 2008). Our large scale synchrony looks like a characteristic of a global brain state, e.g., attentiveness or preparation for movement, rather than encoding of a specific motor behavior. An alternatively explanation is that the entire musculature of the ipsilateral forelimb may be contracting during the reaching behavior, which may be encoded by the observed synchrony within a large cortical area dedicated to the entire forelimb in the PML. In conclusion, further investigation is needed to shed light on how the peripheral sensory and central command information interact at the cerebellar cortex during behavior and if this interaction can be explored with spatiotemporal synchrony patterns of high frequency oscillations in the cerebellar cortex.

### Conflict of interest statement

The authors declare that the research was conducted in the absence of any commercial or financial relationships that could be construed as a potential conflict of interest.
